# Synthesis, Characterization, DNA Interaction, and Antitumor Activities of La (III) Complex with Schiff Base Ligand Derived from Kaempferol and Diethylenetriamine

**DOI:** 10.1155/2014/354138

**Published:** 2014-10-12

**Authors:** Qin Wang, Yu Huang, Jin-Sheng Zhang, Xin-Bin Yang

**Affiliations:** ^1^Rongchang Campus, Southwest University, Chongqing 402460, China; ^2^Pharmacy College, Ningxia Medical University, Yinchuan 750004, China; ^3^School of Chemistry and Material Science, Guizhou Normal University, Guiyang 550001, China

## Abstract

A novel La (III) complex, [LaL(H_2_O)_3_]NO_3_ *·*3H_2_O, with Schiff base ligand **L** derived from kaempferol and diethylenetriamine, has been synthesized and characterized by elemental analysis, IR, UV-visible, ^1^H NMR, thermogravimetric analysis, and molar conductance measurements. The fluorescence spectra, circular dichroism spectra, and viscosity measurements and gel electrophoresis experiments indicated that the ligand **L** and La (III) complex could bind to CT-DNA presumably via intercalative mode and the La (III) complex showed a stronger ability to bind and cleave DNA than the ligand **L** alone. The binding constants (*K*_*b*_) were evaluated from fluorescence data and the values ranged from 0.454 to 0.659 × 10^5^ L mol^−1^ and 1.71 to 17.3 × 10^5^ L mol^−1^ for the ligand **L** and La (III) complex, respectively, in the temperature range of 298–310 K. It was also found that the fluorescence quenching mechanism of EB-DNA by ligand **L** and La (III) complex was a static quenching process. In comparison to free ligand **L**, La (III) complex exhibited enhanced cytotoxic activities against tested tumor cell lines HL-60 and HepG-2, which may correlate with the enhanced DNA binding and cleaving abilities of the La (III) complex.

## 1. Introduction

The metal-based anticancer complexes have attracted many bioinorganic chemists' interest since the success of platinum complexes as anticancer agents [1–3]. Among various metal complexes, La (III) complexes have been intensively investigated due to their more physiological activities and lower toxicities after coordination with ligand. People have paid great interest to synthesis, DNA interaction, and anticancer activity of La (III) complexes in recent years [4–8].

**Scheme 1:**
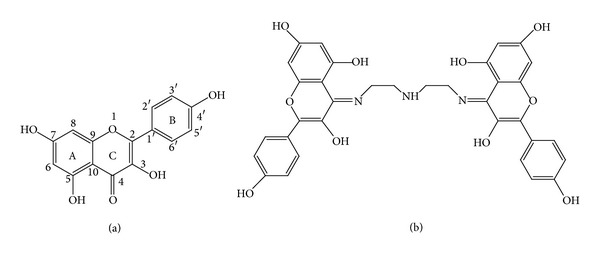
Kaempferol (a) and Schiff base ligand** L** derived from kaempferol and diethylenetriamine (b).

In order to develop novel metal-based anticancer drugs, a new strategy has been adopted in the designs of antitumor coordination compounds based on the traditional Chinese medicines as ligands. A large number of the traditional Chinese medicines have been screened and used for treating and preventing various chronic conditions over long-term folk practice [9, 10]. Flavonoids are a group of naturally occurring compounds that are found in many high plants. Such compounds have been evoked widespread interest in biological and pharmacological activities including antioxidant, anticancer, and antimicrobial, and so forth [11–15]. Moreover, most of flavonoids are strong metal chelators because hydroxy and oxo groups in flavonoid structure have the ability to form complexes with various metal ions, and their biological activities are influenced by the presence of metal ions. The metal complexes derived from such best known flavonoids as quercetin, morin, naringenin, and hesperetin have been extensively studied in recent years [16–21]. Kaempferol (3, 3′, 5, 7-tetrahydroxyflavone), one of the most abundant natural flavonoids, is found in berries, tea,* Brassica* and** Allium** species, and many traditional Chinese herbal medicines (Scheme 1(a)). It is also an attractive reagent due to extensive pharmacological activities [22, 23]. However, to the best of our knowledge, less attention was paid to the DNA interaction and antitumor activities of rare earth metal complexes derived from kaempferol [24]. In this work, we synthesized and characterized a novel La (III) complex with Schiff base ligand derived from kaempferol and diethylenetriamine and focused our attention on comparative studies of DNA binding, DNA cleavage, and* in vitro* antitumor activities of Schiff base ligand** L** (Scheme 1(b)) and its La (III) complex.

## 2. Experiment

All the chemicals were of analytical grades and used without further purification. The concentration of calf thymus DNA (CT-DNA) was determined by UV absorption at 260 nm using a molar absorption coefficient *ε*_260_ = 6600 L mol^−1^ cm^−1^. Purity of CT-DNA was checked by monitoring the ratio of the absorbance at 260 and 280 nm. The solution gave a ratio of >1.8 at *A*_260_/*A*_280_, indicating that DNA was sufficiently free from protein.

Elemental analyses were performed using a Carlo-Elba1106 elemental analytic instrument (Italy). The IR spectra were recorded on a Thermo Scientific Nicolet iS50 FT-IR spectrometer (America) as KBr pellets in the range of 4,000–400 cm^−1^ region. ^1^H NMR spectral data were measured on a Varian INOVA-400 spectrometer (Switzerland) with tetramethylsilane (TMS) as an internal standard. Fluorescence spectra were recorded on an F-7000 spectrophotometer (Japan). Thermogravimetric analysis (TG) was carried out on a SDT Q600 (America) instrument under argon, using *α*-alumina as a reference compound, from room temperature to 800°C at heating rate of 15°C min^−1^. UV-Visible spectra were measured on TU-1901 spectrophotometer (China). Circular dichroism measurements were carried out on a Jasco-815 spectrometer. Conductivity measurement was performed at room temperature using a DDS-11A conductivity meter (China).

### 2.1. Synthesis of Schiff Base Ligand L

Schiff base ligand** L** was synthesized according to the following procedure [25]. Diethylenetriamine (1 mmol) and kaempferol (0.578 g, 2 mmol) were dissolved in ethanol (10 mL). The solution was refluxed for 3 h. The yellow precipitate was filtered off, washed with ethanol, and dried in vacuum. Anal. Calc. for C_34_H_33_N_3_ O_12_: C, 60.44; H, 4.92, N, 6.22%. Found: C, 60.15; H, 4.96; N, 6.16%. IR (KBr, cm^−1^): 3318, 3116, 1659, 1569, 1505, 1312, 1177, 823. ^1^H NMR (d_6_-DMSO 400 MHz) *δ* (ppm): 2.55–2.68 (m, 8H), 6.01 (s, 2H), 6.24 (s, 2H), 6.88-6.89 (d, 4H), 8.01–8.03 (d, 4H).

### 2.2. Synthesis of La (III) Complex

The Schiff base ligand** L** (0.34 g, 0.5 mmol) was dissolved in acetone (20 mL) and triethylamine (130 *μ*L, 1 mmol) was added. After 5 min, La (III) nitrate (0.217 g, 0.5 mmol) was added quickly and the solution was refluxed for 4 h. A brown precipitate was filtered off, washed with ethanol, and dried in vacuum. Anal. Calc. for C_34_H_39_LaN_4_O_19_: C, 43.14; H, 4.15; N, 5.92%. Found: C, 41.67; H, 4.12; N, 5.84%. IR (KBr, cm^−1^): 3380, 2348, 1606, 1384, 1265, 1172, 887, 511. ^1^H NMR (d_6_-DMSO 400 MHz) *δ* (ppm): 2.66–2.85 (m, 8H), 5.69 (s, 2H), 5.93 (s, 2H), 6.84–6.87 (d, 4H), 8.38–8.42 (d, 4H).

### 2.3. DNA Binding and Cleavage Experiments

Fluorescence quenching experiments were carried out by adding the increasing amounts of compounds (0, 30, 60, 90, 120, and 150 *μ*M) to EB-DNA system (C_EB_ = 50 *μ*M, C_DNA_ = 200 *μ*M, 0.1 M Tris-HCl buffer solution, pH = 7.4). The fluorescence spectra were measured at three different temperatures (298, 303, and 310 K). Emission spectra were carried out in 3 mL quartz cuvette with 339 nm excitation light, and emission was measured at 590 nm.

Viscosity experiments were carried on an Ubbelohde viscometer, immersed in a thermostated water bath maintained at 25°C. The concentration of DNA was 100 *μ*M in buffer solution (0.1 M Tris-HCl, pH 7.4). Data were presented as (*η*/*η*_0_)^1/3^ versus the concentration of the complexes, where*η* is the viscosity of DNA in the presence of compounds (4, 8, 12, 16, 20, 24, and 28 *μ*M) and *η*_0_ is the viscosity of DNA alone.

Circular dichroism (CD) absorption spectra of DNA were measured in buffer solution (0.1 M Tris-HCl, pH 7.4) at a 50 nm/min scan rate in the wavelength range from 220 to 300 nm, with 100 *μ*M DNA in the absence and presence of the compounds (20 *μ*M). All experiments were done using a quartz cell of 1 mm path length. The spectra were recorded at 25°C after the compounds had been incubated with CT-DNA for 4 h at 37°C.

Plasmid DNA (pUC 19) cleavage activity of the compounds was monitored by using agarose gel electrophoresis. In a typical experiment, supercoiled DNA (pUC 19) (50 g/mL, 5 *μ*L) in Tris-HCl (100 mM, pH 7.4) was treated with concentrations (100 *μ*M) of different compounds, followed by dilution with the Tris-HCl buffer to a total volume of 20 *μ*L. The samples were then incubated at 37°C and loaded on a 0.1% agarose gel containing 1.0 g/mL of ethidium bromide. Electrophoresis was carried out at 80 V for 40 min in TAE buffer and run in duplicate. Bands were visualized by UV light and photographed.

### 2.4. *In Vitro* Cytotoxicity Assay

Cytotoxicities of all the compounds against HL-60 (human leukemia cell) and HepG-2 (liver hepatocellular carcinoma cell) were determined by WST-8 assay (WST-8 sodium2-(2-methoxy-4-nitro-phenyl)-3-(4-nitrophenyl)-5-(2, 4-disulfophenyl)-2H-tetrazolium) with cell counting kit-8 (CCK-8). The cells were plated in 96-well culture plates at density of 1 × 10^4^ cells per well and incubated for 24 h at 37°C in a water-atmosphere (5% CO_2_). The tested compounds with various concentrations (0, 12.5, 25, 50, and 100 *μ*M) were obtained by dissolving them in DMSO and diluting them with culture medium (DMSO final concentration < 1%). Then the diluted solution of compounds was treated with the cells for 24 h at 37°C in a 5% CO_2_ incubator. After that, 10 *μ*L of a freshly diluted CCK-8 solution (5 mg/mL in PBS) was added to each well for 2 h. The cell survival was evaluated by measuring the absorbance at 450 nm. The IC_50_ which inhibits growth of 50% of cells relative to nontreated control cells was calculated as the concentration of tested compound. All experiments were carried out in triplicate.

## 3. Results and Discussion

### 3.1. Characterization

The molar conductivity of the La (III) complex is 115 S cm^2 ^mol^−1^ in DMF solution, indicating the electrolytic nature of the complex. The TG curve of complex shows a series of weight loss upon heating. The first weight loss 5.88% (calculated 5.71%) is probably assigned to three cocrystallized water molecules by 120°C. The second weight loss 5.56% (calculated 5.71%) in the range of 120–260°C is coincided with the three coordinated water molecules. The elemental analysis, molar conductivity, and TG indicate that formula of the La (III) complex conforms to [LaL(H_2_O)_3_]NO_3_ *·*3H_2_O.

On the comparison of the IR spectra of the La (III) complex with ligand** L**, the important information on coordination reaction was provided. The characteristic stretching (C=N) mode of ligand** L** occurs at 1660 cm^−1^, while, due to the formation of complex, this band appears at about 1606 cm^−1^. It can be suggested that the La (III) coordination occurs through the imine nitrogen atom. In addition, a characteristic vibration band of free nitrates was observed at about 1384 cm^−1^ in the spectrum of the complex [21]. The La (III) complex and ligand** L** were also examined by H NMR spectra, and the chemical shift differences were shown in Table 1. The complex exhibited upfield shifts due to electron transfer from the imine nitrogen and the hydroxyl oxygen atom to La (III). There was a negative shift effect for the H_6_ and H_8_ of ring A of the complex and a positive shift effect for the H_2′,6′_ of ring B of the complex existed. The results suggested that ligand** L** could be chelated to La (III) ion via both the imine nitrogen and 3-OH of C ring on the basis of the H^1^ NMR data [9, 26].

**Figure 1:**
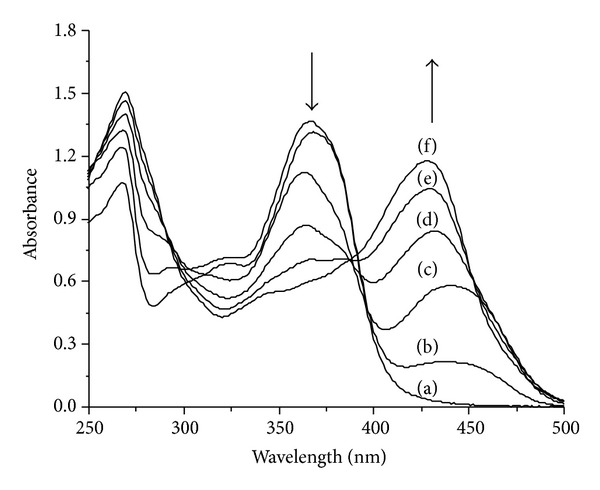
Absorption spectra of ligand** L** in ethanol in the presence of La (III). The molar ratios [La(NO_3_)_3_]/[ligand] = 0 (a), 0.2 (b), 0.4 (c), 0.6 (d), 0.8 (e), and 1.0 (f).

The interactions between ligand** L** and the La (III) ion were also studied by absorption spectra in order to further understand structure information of the complex. The ligand** L** absorbed with maxima at 368 nm (band I) and 269 nm (band II). Band I is related to ring B (cinnamoyl system) and band II to ring A (benzoyl system) [27, 28]. Absorption spectra of ligand** L** in the ethanol solution with different concentrations of La(NO_3_)_3_ are shown in Figure 1. The intensity of ligand** L** (band I) decreased gradually with addition of La (III) to the solution and a new absorbance peak appeared at 427 nm. The results indicated that ligand** L** could form complex with La (III). The appeared new peak at 427 nm suggested that La (III) had bonded to 3-hydroxyl and C=N of ring C. Band I bathochromic shift can be explained by the interaction of La (III) with the 3-hydroxyl group of ring C producing an electronic redistribution between ligand** L** and La (III), which resulted in an extended *π*-bonding system. The 5-OH group is not involved due to lesser proton acidity and the steric hindrance caused by the first complexation [18, 24].

**Figure 2:**
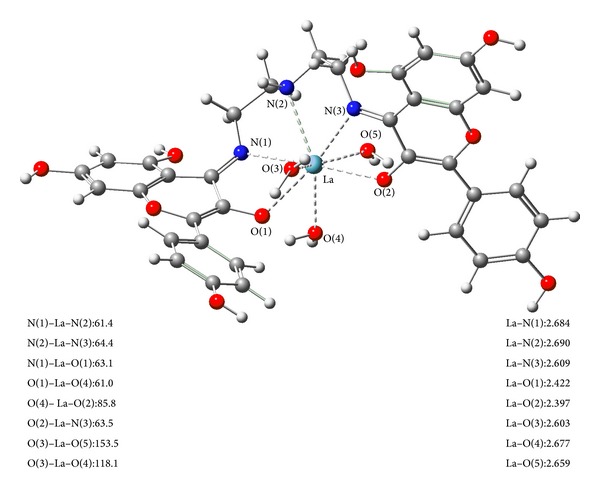
The optimized structure of the La (III) complex. The sky blue ball is for La atom, the red balls are for oxygen atoms, the blue balls are for nitrogen atoms, the gray balls for carbon atoms, and the white balls are for hydrogen atoms. Bond lengths are in angstrom and bond angles are in degree.

Since no single crystals suitable for X-ray determination could be isolated, structural information for the La (III) complex was also obtained from the B3LYP optimization calculations, as shown in Figure 2. Theory calculation result was in good agreement with above studies.

### 3.2. DNA Binding and Cleavage Studies

**Figure 3:**
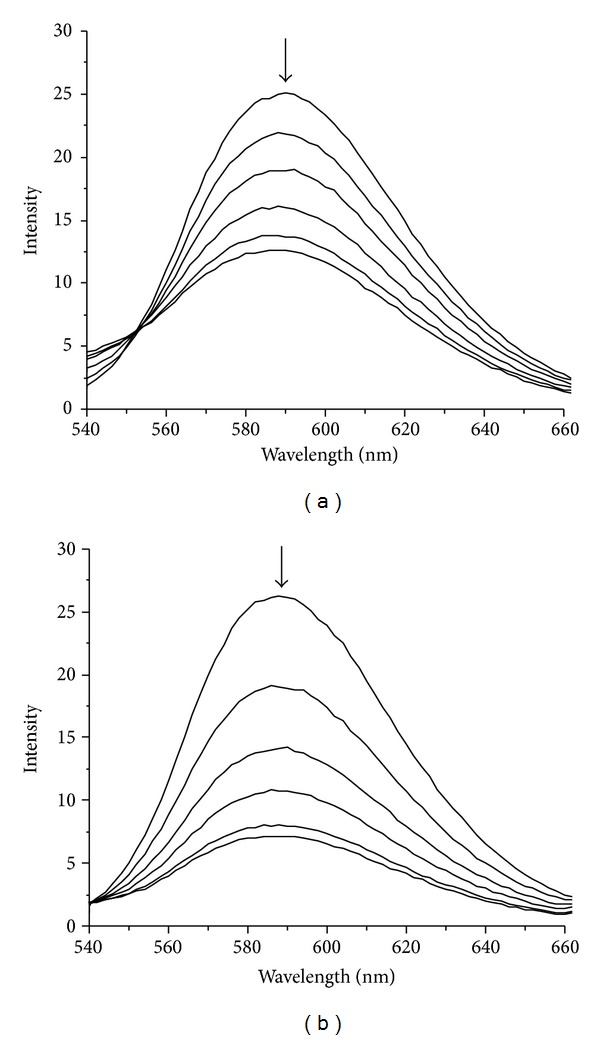
Emission spectra of DNA-EB with various concentrations of ligand** L** (a) and La (III) complex (b) at 298 K, [EB] = 50 *μ*M, [DNA] = 200 *μ*M, and [compound] = 0, 30, 60, 90, 120, and 150 *μ*M, respectively. The arrow shows the intensity changes on increasing the compound concentration.

Since DNA is the primary intracellular target of anticancer drugs, the interaction studies of drugs with DNA are very important in the development of new therapeutic reagents. Binding abilities of ligand** L** and La (III) complex to CT-DNA were investigated by EB-competitive binding experiments. Ethidium bromide (EB) has weak fluorescence, but its emission intensity in the presence of DNA could be greatly enhanced because of its strong intercalation between the adjacent DNA base pairs on the double helix. It was previously reported that this enhanced fluorescence could be quenched, at least partly by the addition of a competing agent [29, 30]. The emission spectra of EB bound to CT-DNA in the absence and presence of ligand** L** and La (III) complex are shown in Figure 3, respectively. The addition of ligand** L** and La (III) complex to DNA solutions pretreated with EB caused appreciable decrease in the emission intensity, which indicated that ligand** L** and La (III) complex competed with EB in binding to DNA through intercalative mode.

**Figure 4:**
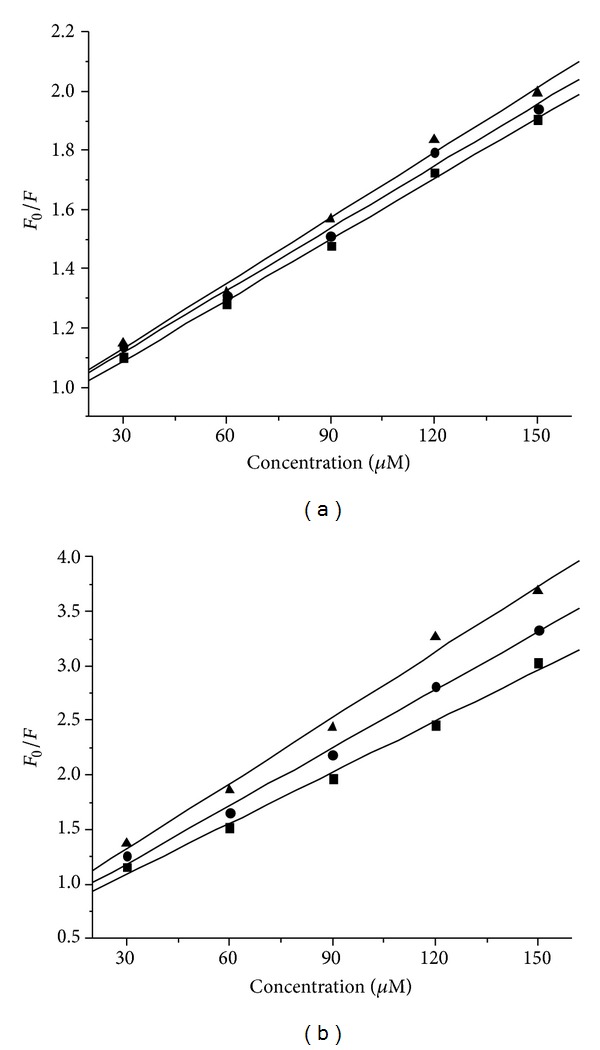
Stern-Volmer plots for the fluorescence quenching of DNA-EB system by ligand** L** (a) and La (III) complex (b) at different temperatures, (▲) 298 K; (●) 303 K; (■), 310 K.

The mechanisms of fluorescence quenching are classified as either dynamic or static quenching, which can be distinguished by their different dependences on temperatures. Generally, the fluorescence quenching was analyzed according to the Stern-Volmer equation [31] as follows:

(1) $$\begin{array}{c} \frac{F_{0}}{F}=1+K_{\text{SV}}\left[Q\right]=1+K_{q}\tau_{0}\left[Q\right], \end{array}$$

where *F*_0_ and *F* are the fluorescence intensities in the absence and presence of the compound, respectively. *K*_SV_ is a linear Stern-Volmer quenching constant, *K*_*q*_ is the quenching rate constant, *τ*_0_ is the average lifetime of molecules in the absence of quencher and its value is about 10^−8^ s [32], and [*Q*] is the concentration of the compound. The Stern-Volmer plots of *F*_0_/*F* versus [*Q*] at three different temperatures are presented in Figure 4. The values of *K*_SV_ are listed in Table 2. The values of *K*_SV_ decreased with the increasing temperatures, and the values of *K*_*q*_ were much larger than the limiting diffusion constant of the biomacromolecules (2.0 × 10^10^ L mol^−1^ s^−1^) [33]. The results suggested that the quenching mechanism of the system was a static quenching process.

**Figure 5:**
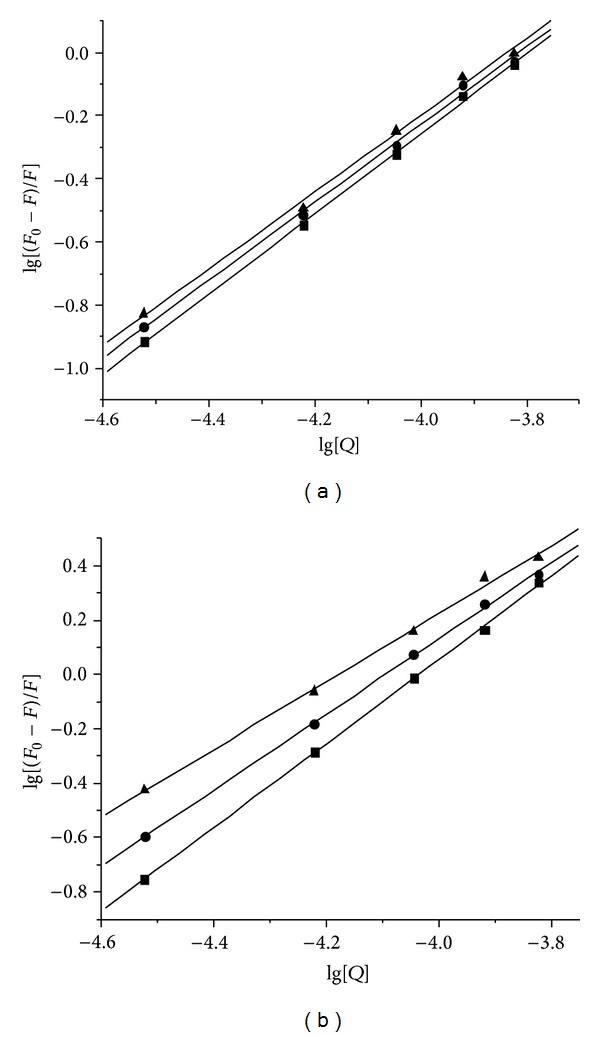
Plots of lg[(*F*_0_ − *F*)/*F*] versus lg[*Q*] for the fluorescence quenching of DNA-EB system by ligand** L** (a) and La (III) complex (b) at different temperatures, (▲) 298 K; (●) 303 K; (■), 310 K.

For a static quenching process, the binding constant (*K*_*b*_) and the number (*n*) of binding sites for ligand** L** and La (III) complex with DNA can be determined by the following equation [34]:

(2) $$\begin{array}{c} \text{ lg }\left[\frac{F_{0}-F}{F}\right]=\text{lg}\;K_{b}+n\;\text{lg}\left[Q\right], \end{array}$$

where *K*_*b*_ and *n* are the binding constant and the number of binding sites in base pairs, respectively. The plots of lg[(*F*_0_ − *F*)/*F*] versus lg[*Q*] were showed in Figure 5, and the values of *K*_*b*_ and *n* were listed in Table 3. The binding constant *K*_*b*_ increased with the increasing temperature, indicating that rising temperature contributed to the binding of ligand** L** and La (III) complex with DNA and the La (III) complex has much stronger binding ability than ligand** L**. Compared to the binding constant of EB with DNA (5.16 × 10^5^ L mol^−1^) [35], the ligand** L** and La (III) complex could compete against EB and replace the intercalated EB from the DNA-EB complex system.

**Figure 6:**
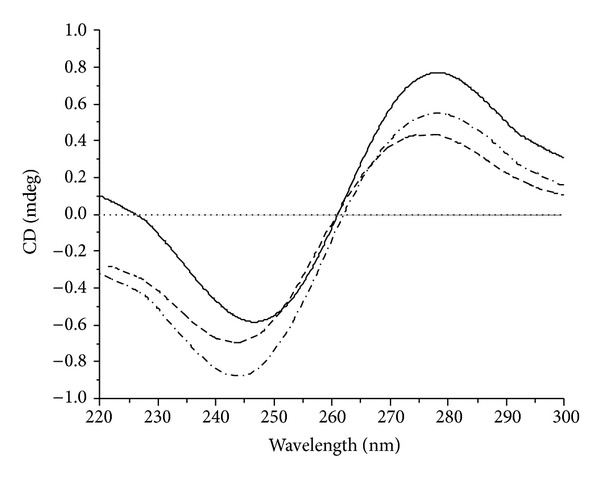
CD spectra of CT-DNA (100 *μ*M) in the absence and presence of the compounds (20 *μ*M). The solid line is for DNA alone, the short dash line for DNA + ligand** L**, and the dash line for DNA + La (III) complex.

CD spectroscopy is useful in monitoring the conformational variations of DNA in the presence of drug in solution. CD spectral variations of CT-DNA in the absence and presence of the ligand** L** and La (III) complex were given in Figure 6, respectively. The free CT-DNA exhibits two conservative CD bands, a positive band at 275 nm due to base stacking and a negative band at 245 nm due to right-handed helicity which is characteristic of DNA in the right-handed B form [36]. When the ligand** L** and La (III) complex are incubated with CT-DNA, the CD spectra of CT-DNA undergo significant variations in both positive and negative bands in intensity. The results suggested that the ligand** L** and La (III) complex could induce disturbance on DNA base stacking and on DNA right-handed helicity. The arrangement of the DNA bases is somewhat altered, and the characteristics of the B form CD spectrum are still conserved. These changes further confirm the intercalative binding of the ligand** L** and La (III)complex with CT-DNA [37, 38].

**Figure 7:**
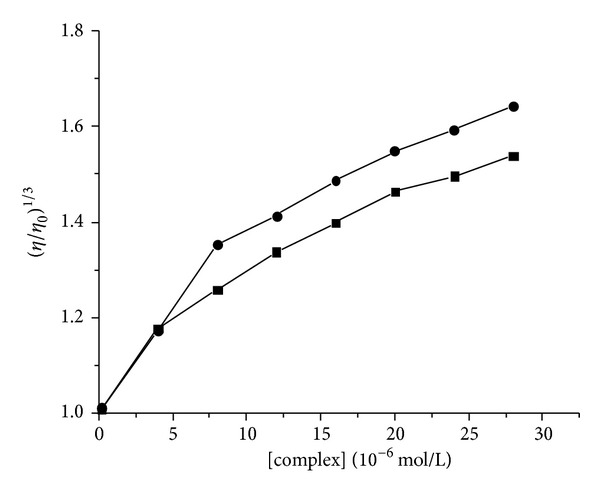
Effects of increasing concentrations of ligand** L** (●) and La (III) complex (■) on the viscosity of CT-DNA, [DNA] = 100 *μ*M, [compound] = 0, 4, 8, 12, 16, 20, 24, and 28 *μ*M, respectively.

In addition to spectroscopic data, the viscosity measurement of CT-DNA is also an effective tool to study the binding mode of the compound to DNA in the absence of crystallographic structural data and NMR. A classical intercalative mode would cause elongation of DNA polymer as base pairs were separated to accommodate the bound compound, resulting in an increase in its viscosity. In contrast, a partial and/or nonclassical intercalation of compound may bend (or kink) the DNA helix resulting in a decrease in its effective length, reducing its viscosity concomitantly [39, 40]. The effects of ligand** L** and La (III) complex on the viscosity of CT-DNA were given in Figure 7. It could be seen that the relative viscosity of DNA increased steadily with increasing concentrations of ligand** L** and La (III) complex, respectively. The results revealed that ligand** L** and La (III) complex could intercalate between adjacent DNA base pairs, causing an extension in the helix and increase in viscosity of the DNA. The results were consistent with the above spectral results.

**Figure 8:**
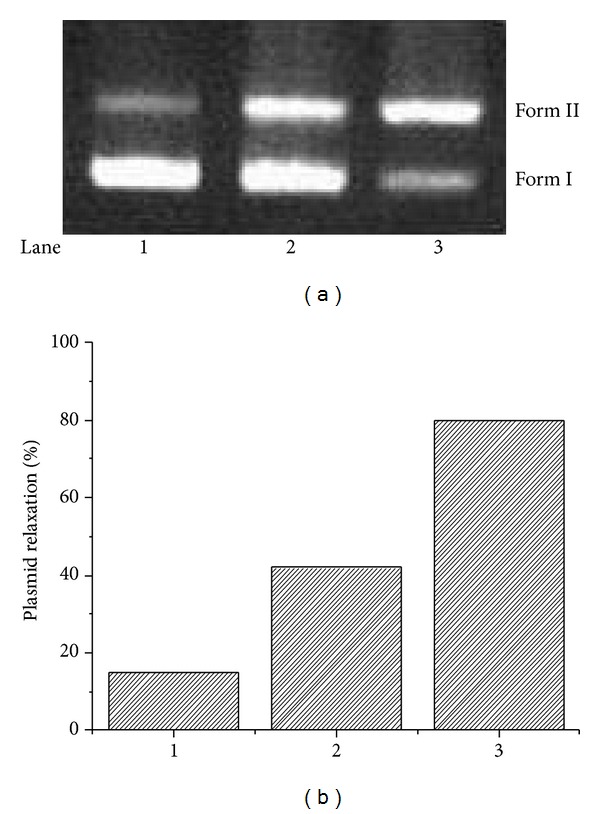
Effect of different compounds (100 *μ*M) on the cleavage reactions of pUC 19 DNA (12.5 *μ*g/mL) in a Tris-HCl buffer (100 mM, pH 7.4) at 37°C for 4 h. (a) Agarose gel electrophoresis diagram: lane 1, DNA control; lane 2, ligand** L**; lane 3, La (III) complex. (b) Quantitation of % plasmid relaxation (Form II %) relative to plasmid DNA per lane.

The abilities of ligand** L** and La (III) complex in inducing DNA cleavage were further investigated by gel electrophoresis using plasmid DNA (pUC 19) in the absence of external reagent or light. The DNA cleavage was analyzed by monitoring the conversion of supercoiled circular DNA (form I) to nicked circular DNA (Form II) under physiological conditions. The amounts of strand scission were assessed by an agarose gel electrophoresis.  Figure 8 showed the relative cleavage efficiency of ligand** L** and La (III) complex. It was obvious that La (III) complex showed more efficient DNA cleavage ability than ligand** L**.

### 3.3. Antitumor Activity

To compare antitumor activities of La (III) complex, ligand** L,** and La (III) nitrate, the cytotoxic activities of all compounds were tested by means of CCK-8 assay against the proliferation of HL-60 and HepG-2 cell lines. The results of cytotoxic activities against tumor cells are expressed as IC_50_ values and are presented in Table 4. The La (III) complex showed higher cytotoxic activities than ligand** L** and La (III) nitrate according to the IC_50_ values of the tested compounds in Table 4, which may be attributed to the extended *π*-bonding system resulting from the chelation of the metal ion with the ligand** L** [20]. Furthermore, the La (III) complex was more potent than the 5-fluorouracil (5-FU) against the HL-60 tumor cell line.

## 4. Conclusions

In summary, a novel La (III) complex with Schiff base ligand derived from kaempferol and diethylenetriamine has been prepared and characterized. The ligand** L** and its La (III) complex interacted with DNA by intercalation mode, and La (III) complex has much stronger DNA binding and cleaving abilities than ligand** L**. The results of* in vitro* cytotoxic activities against HL-60 and HepG-2 cell lines indicated that the La (III) complex exhibited more effective cytotoxic activities than the ligand** L**, which may correlate with the enhanced DNA binding and cleavage abilities of the La (III) complex.

## Figures and Tables

**Table 1:** ^
1^H NMR chemical shift differences between **L** and the complex.

Hydrogen site	*δ* (**L**)	*δ* (complex)
6	6.01	5.69
8	6.23	5.92
3′, 5′	6.88–6.90	6.84–6.86
2′, 6′	8.01–8.03	8.38–8.42

**Table 2:** The Stern-Volmer quenching constants of system at various temperatures.

Compound	*T* (K)	*K*_SV_ (10^4^ L mol^−1^)	*K*_*q*_ (10^12^ L mol^−1^ s^−1^)	*R*
Ligand **L**	298	0.734	0.734	0.9966
	303	0.697	0.697	0.9956
	310	0.684	0.684	0.9985

La complex	298	2.01	2.01	0.9950
	303	1.77	1.77	0.9976
	310	1.56	1.56	0.9966

*R* is the correlation coefficient.

**Table 3:** The binding constants and binding sites at various temperatures.

Compound	*T* (K)	*K*_*b*_ (10^5^ L mol^−1^)	*n*	*R*
Ligand **L**	298	0.454	1.21	0.9983
	303	0.504	1.23	0.9985
	310	0.659	1.27	0.9995

La complex	298	1.71	1.25	0.9984
	303	5.33	1.40	0.9996
	310	17.3	1.54	0.9998

*R* is the correlation coefficient.

**Table 4:** IC_50_ values of the tested compounds against tumor cell lines.

Cell line	IC_50_ value (*μ*M)			
	La (III) complex	Ligand **L**	La (III) nitrate	5-FU
HL-60	22.37 ± 0.93	32.49 ± 1.12	>100	37.59 ± 1.51
HepG-2	17.12 ± 1.06	24.39 ± 1.20	47.16 ± 2.65	6.78 ± 0.65

5-FU was used as control.
